# COVID-19 Serology Data Provide Guidance for Future Deployments of Convalescent Plasma

**DOI:** 10.1128/mbio.00428-23

**Published:** 2023-04-11

**Authors:** David Sullivan, Arturo Casadevall

**Affiliations:** a Department of Molecular Microbiology, Johns Hopkins School of Public Health, Baltimore, Maryland, USA

**Keywords:** COVID-19, SARS-CoV-2, convalescent plasma

## Abstract

Measurement of antibody content and function after a viral illness is important for diagnosis and selection of the best convalescent plasma (CP) units for passive immunization. Zhang et al. (mBio 14:e03523-22, 2013, https://doi.org/10.1128/mbio.03523-22) analyzed over 19,000 coronavirus disease 2019 (COVID-19) CP (CCP) samples from the early days of the COVID-19 pandemic and reported a moderately strong correlation between antibody amount and neutralizing titer. Strikingly, about one-third of the samples had little or no neutralizing activity. The results provide a detailed glimpse of the humoral immune response to severe acute respiratory syndrome coronavirus 2 (SARS-CoV-2) in immunologically naive humans and reveal major differences in the quality of CP units collected for passive therapy before antibody screening. Heterogeneity in CCP quality undoubtedly contributed to the variable therapeutic efficacy. Analysis of the COVID-19 serology data suggest that, for the next infectious disease emergency, the best approach after quick establishment of methods for robust antibody-level stratification would be to use CP units in the top quintile of antibody content and neutralizing capacity.

## COMMENTARY

When the coronavirus disease 2019 (COVID-19) pandemic struck the United States in the spring of 2020, the country responded by deploying convalescent plasma (CP) as an emergency interim therapy, first under the Expanded Access Program (EAP) (1) and later under emergency use authorization (EUA), resulting in treatment of >500,000 hospitalized patients (2). In those terrible early days of the COVID-19 pandemic, there were no specific therapies available, and COVID-19 CP (CCP) was deployed based on historical knowledge of efficacy against prior epidemics. At the time, a paucity of serological tests precluded determination of antibody presence or levels in real time, with qualifications based on documented severe acute respiratory syndrome coronavirus 2 (SARS-CoV-2) tests and COVID-19 symptoms. A variety of commercial serological tests were later allowed with little validation because of the emergency nature of the pandemic. Now, Zhang et al. (3) have analyzed >19,000 CCP donor samples from March and August 2020, providing a detailed look at CCP antibody quantity and quality in the early days of the pandemic. This information provides a retrospective analysis of the functional content of CCP used and informs best practices for the deployment of CP in future emergencies with other respiratory viruses.

Before considering the results of Zhang et al. (3), it is worthwhile to review the anatomy of SARS-CoV-2 and its interactions with specific antibodies. The SARS-CoV-2 virion has about 100 spike protein molecules, 2,000 membrane proteins, and about 20 envelope proteins exposed on a 31,314-nm^2^ coronal viral surface (4). The 200-amino acid receptor binding domain (RBD) represents 15% of the spike protein content but accounts for the most potent virus-neutralizing antibodies; antibodies to RBD epitopes are better neutralizers than are antibodies to epitopes on the stalk of the spike. Antibodies raised solely to the spike protein, such as those elicited by infection vaccines or included in monoclonal antibody preparations, are sufficient to prevent infection and halt disease progression through virus neutralization. While non-spike viral antibodies can contribute to virus neutralization, these are not necessary or sufficient. In addition, nonantibody innate and acute-phase proteins also make minor contributions to early, variable viral clearance, including mannose-binding lectins, membrane C-type lectins, the inflammasome, and endosomal viral nucleic acid recognition proteins such as the cyclic GMP-AMP synthase-stimulator of interferon genes (STING) pathway, which also are insufficient for virus neutralization (5).

Good news and bad news for CP therapeutic use are evident in the results. The good news is that there is a reasonably good correlation between antibody quantitative content and the neutralizing activity of CCP, a finding that was hinted at by earlier smaller studies (6). This is important because it implies that it should be possible to select high-quality CCP units using antibody titers alone with a good probability that they would contain neutralizing antibody. However, a caveat in this insight is that the correlation between antibody content and neutralization was strongest for units in the higher percentiles. After validation of assays with 420 plasma donors, Zhang et al. (3) found strong correlations of virus-neutralizing activities and serological levels for the upper one-half of the 19,000 plasma donors but weaker correlations for the lower 30%. Strikingly, nearly 10% of samples were not seropositive in the RBD enzyme-linked immunosorbent assay (ELISA), while 8% (1,570 samples) did not have detectable virus-neutralizing activity. Another 25% of CCP units had low virus-neutralizing activity, with two-thirds possessing moderate to high virus-neutralizing activity. Interestingly, about 10% of the samples had indeterminate IgG RBD or nucleocapsid (N) antibodies. It is noteworthy that the antibody content assay measured only IgG, although IgA and IgM also neutralize SARS-CoV-2; this might have reduced the correspondence between antibody content and neutralization capacity for some samples. Indeed, neutralization by IgA, IgM, or S1-specific IgG antibodies was proved in a small subset of 25 samples without measurable antibodies to RBD or N. Hence, it is good news to know that, early in the pandemic, there were many individuals with high-titer responses who were excellent donors for CCP and many COVID-19 patients were treated with high-quality CCP. The bad news is that about one-third of the CCP samples had little or no neutralizing activity, which suggests that such units are inadequate for passive therapy. Hence, selection of CCP for therapy without measuring antibody titers, as was done in the early days of the pandemic, would result in about one-third of patients receiving inadequate therapy.

It is noteworthy that Zhang et al. (3) reported that the upper 12% of CCP samples from infections with the WA-1 SARS-CoV-2 strain full virus neutralized Omicron variants that appeared 18 months later. This finding anticipates later reports that individuals who received mRNA vaccines and had subsequent COVID-19 breakthrough infections with pre-Omicron SARS-CoV-2 variants had plasma with high titers of neutralizing antibodies to Omicron (7). This is a fascinating example of how the immune system diversifies its antibody response such that infection with one variant can produce response-neutralizing antibodies against a future variant virus, and it helps explain why COVID-19 from infection with early SARS-CoV-2 variants conferred protection against latter variants.

The results from Zhang et al. (3) also allow some estimates of the antibody amounts transfused with the units available early in the pandemic. The RBD ELISA quantified RBD antibodies at 54 μg/mL (95% confidence interval [CI], 54 to 55 μg/mL) for the nearly 19,000 samples, which was similar to the value of 57 μg/mL in the validation set of 420 samples. Since total IgG is about 10 mg/mL for 3,000 mL adult plasma volume, this implies that about 0.5% of the 30 g of total IgG, or 150 mg, was directed to the spike RBD. Avogadro’s number predicts 10^20^ total antibodies, with 0.5 × 10^18^ (one-half of a quintillion) SARS-CoV-2-specific antibodies. It could be further estimated that the total antibody to full-length spike antibodies would approximate twice the RBD-specific antibody. For a 200-mL dose of plasma, this translates to virus-specific antibodies of 10 mg RBD IgG or 20 mg full-length spike IgG, which is 15 to 100 times less than the virus-specific dose of monoclonal antibodies, with the caveat that the latter target a single epitope, while CCP is polyclonal and thus targets multiple epitopes in the RBD. The good news is that antibody levels on a population level are at least 10-fold higher than those observed by Zhang et al. (3) for individuals who received repeated vaccinations and had SARS-CoV-2 breakthrough infections, suggesting closer to 200 mg of SARS-CoV-2 in 200 mL of therapeutic plasma. Antibody levels 2 to 4 times CP equivalents induced to full-length spike proteins by vaccines prevent most infections and reduce the need for hospitalizations but have no impact on patients already hospitalized (4, 8). Neutralizing monoclonal antibodies doses have 15 to 20 times the amount of specific antibody for spike protein, compared to CP equivalents (9, 10), and they also prevent infection (10, 11), reduce outpatient progression to hospitalization (12), and have a smaller impact on already hospitalized patients (13–15). In paired outpatient studies, pre-Alpha CCP was not shown to prevent infection (16), while the same donor pools with equivalent antibody levels reduced the risk of hospitalizations by >50% (17) and by 80% when given within 5 days after symptom onset, as was tested for many monoclonal antibodies (18). High-titer CCP reduced deaths among hospitalized patients (19).

We now know that CCP is effective when administered early in the course of disease using units with sufficient titers to mediate a pharmacological effect (17, 20). The temporal requirement is dictated by the mechanism of action of CCP, which is primarily an antiviral therapy. Like all antiviral therapies, CCP works best when given early during the viral phase of disease and before the onset of life-threatening pulmonary inflammation. In the fourth year of the pandemic, most of humanity has some immunity to SARS-CoV-2 because of vaccination or infection, including humoral immunity. Therefore, the importance of CCP as a therapy has declined for immunocompetent individuals, most of whom now have their own IgG, but remains important for the treatment of immunocompromised individuals, who often lack antibody to the virus (21).

**FIG 1:**
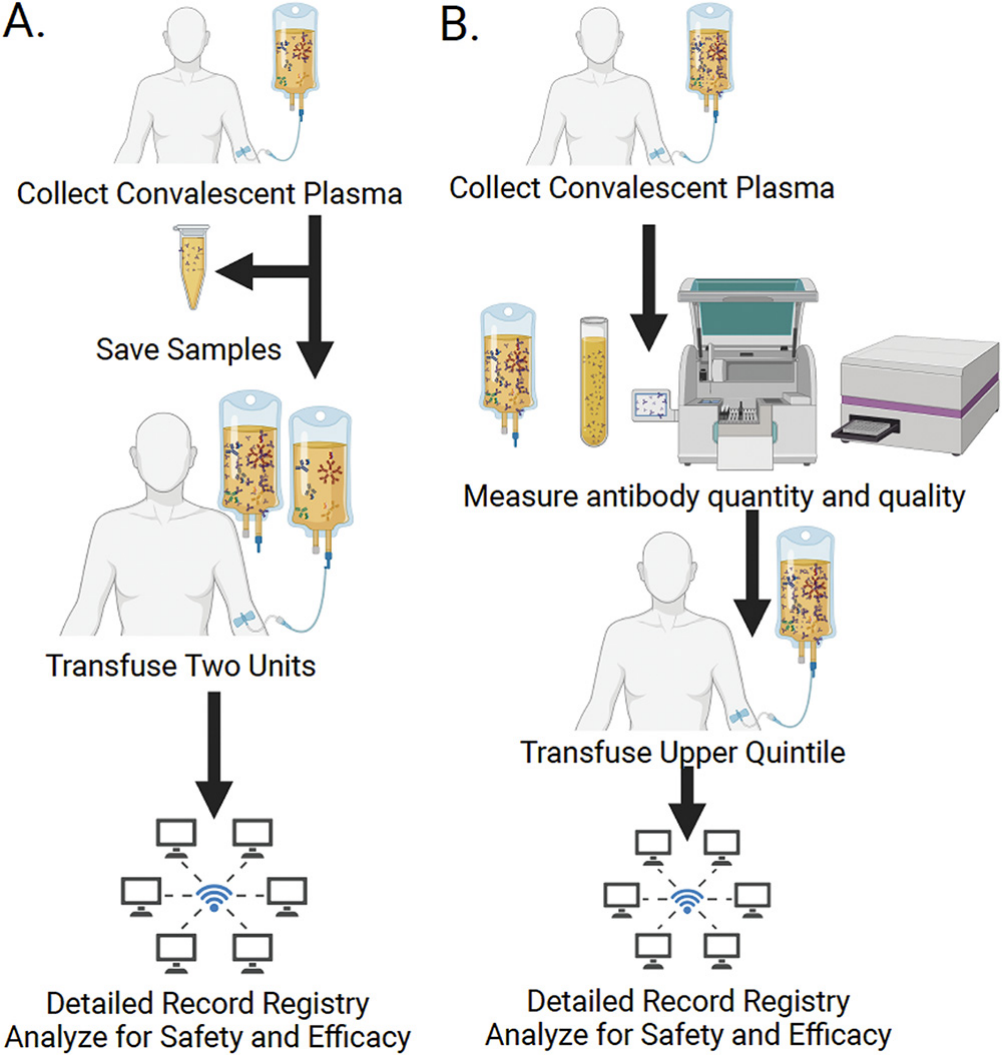
Scheme for using CP in a future infectious disease emergency. (A) Deployment of CP in the absence of assays to measure the antibody concentration and neutralizing activity. In the absence of assays, CP can be deployed based on clinical history of having survived the disease, as was performed early in the COVID-19 pandemic in the United States. In that situation, samples of transfused CP should be saved for later analysis to correlate antibody amounts with clinical efficacies. Transfusing 2 units increases the likelihood of administering 1 unit with high antibody content. The alternative of pooling units is not feasible without regulatory changes and brings possible complications with regard to blood group matching. (B) Deployment of CP when assays to measure the antibody concentration and neutralizing activity are available. In this situation, it should be possible to measure the antibody contents of several hundred units to establish the range of antibody concentrations and then pick the top quintile for clinical use. In both types of situations, registries can be used to obtain evidence for safety and efficacy.

In the first 23 years of the 21st century, humanity has already confronted at least seven major viral outbreaks, as illustrated by the emergence of SARS-CoV, Middle East respiratory syndrome (MERS)-CoV, influenza A virus subtype H1N1, Zika virus, Ebola virus, SARS-CoV-2, and monkeypox virus. CP was used or considered for all these viruses (22–24). Whenever a new viral threat emerges, CP is always considered, since this is a venerable therapy that was first used successfully in the 1918 influenza pandemic (25). Historically, CP was always used in emergency situations; consequently, it was not possible to carry out careful serological and clinical studies during the crisis. The COVID-19 pandemic has been sufficiently protracted that a voluminous trove of data has now been assembled that conclusively establishes the conditions under which CP is expected to be effective, namely, when used early in the disease with high-titer CP. At the time of this writing, we are living through a worldwide avian influenza virus H5N1 outbreak, and there is fear of a spillover to human populations. Should that calamity occur, it can be anticipated that H5N1 CP will be used until better therapies are developed. The very detailed serological analysis of CP from the early days of the pandemic provided by Zhang et al. (3) suggests a roadmap for future deployments of CP, whether against influenza or against another coronavirus, and, in that eventuality, suggest the following best practices from the COVID-19 experience (Fig. 1).

First, CP can be expected to show great variability in total antibody levels and neutralizing titers. Hence, using CP units without measuring these parameters will result in a significant proportion of recipients receiving units with little or no antibody content. This occurred not only in the early days of the COVID-19 pandemic but also in other infectious disease emergencies when CP was used, such as the recent West African Ebola virus outbreak (26). To avoid this, it is imperative to rapidly develop assays for quantitation of antibody amounts and neutralizing capacities, which would allow the selection of units with the greatest amounts of antibody possible. Until such screening procedures are in place, physicians could increase the odds of providing sufficient antibody by transfusing multiple CP units from different donors. Given the fact that studying a few hundred samples mirrored the results for thousands reported by Zhang et al. (3), it should be possible to rapidly screen a defined set of CP samples to establish the range of antibody concentrations and to select units with the greatest antibody contents.

Second, the effectiveness of CP can be expected to be highly dependent on meeting the three principles of antibody efficacy (23), namely, that CP units (i) have specific antibody, (ii) are present in sufficient amounts, and (iii) are used early in the course of disease. Clinical trial design should incorporate these principles and test CP early in the disease, preferably in outpatients before the disease has progressed to a life-threatening stage. Although testing CP efficacy in outpatients is more complicated than inpatient efficacy studies, the experience from COVID-19 shows how this can be done (27).

Third, until clinical dose-response efficacy data are available, clinicians can anticipate that the antibody content and neutralizing capacity data reported by Zhang et al (3) will be relevant, showing a diamond-shaped pattern for antibody levels. The lower 40% should not be considered for treatment. The middle 40 to 60% will require close variant matching for activity and high volume and will be diluted to levels below the geometric mean of CCP. The upper 60 to 80% might be utilized when other measures are scarce. The upper 20% is the best CP and should be used preferentially in both clinical use and efficacy trials. These high-titer units are more likely to provide protection when diluted 10- to 20-fold into the 3-L plasma volume by providing neutralization capacity and SARS-CoV-2 antibody levels above the geometric mean of CCP levels.

Fourth, until clinical efficacy data from randomized clinical trials (RCTs) are available during the next pandemic, the establishment of registries that track usage and clinical outcomes, such as the EAP, can provide valuable information on safety (28) and dose-response relationships (19), and CP samples should be saved for retrospective analysis, as was done by Zhang et al. (3). Although this approach was criticized during the COVID-19 pandemic because it was thought to interfere with RCTs, the fact is that several trials were completed in the United States while CCP was under EUA. Hence, registries and RCTs can be used simultaneously and provide complementary information on safety and efficacy.

Finally, we consider the most important question in the use of CP: what is the range of antibody levels that define therapeutically effective CP? Mathematical modeling of vaccines, monoclonal antibodies, and CCP have also utilized the ratiometric CP for efficacy. Antibody levels that reduce 50% of hospitalizations reduce only about 15% of infections (29, 30). The goal for therapeutic plasma is to have antibody levels that remain above the mean levels after 20-fold dilution. The top 20 to 30% of units will retain activity against future variants.
